# Why are pro-poor exemption policies in Tanzania better implemented in some districts than in others?

**DOI:** 10.1186/1475-9276-12-80

**Published:** 2013-09-26

**Authors:** Stephen O Maluka

**Affiliations:** 1 Institute of Development Studies, University of Dar es Salaam, Box 35169, Dar es Salaam, Tanzania

**Keywords:** Exemption policy, Policy implementation, Health facility committees, Tanzania

## Abstract

**Background:**

Like other African countries, Tanzania has in recent years, been implementing various exemptions and targeting programmes to protect and ensure equitable access to health care by poorer segments of the population. A body of evidence indicates that exemption policies, while potentially effective in principle, are ineffective in implementation. However, there is evidence that some districts, despite the challenges, perform better than others in terms of identifying the poor and allocating funds for the poor and vulnerable groups.

**Methods:**

Drawing from the review of minutes, health facility visits, and key informant interviews with the community representatives and the district health managers, the study explored why exemption policies in Tanzania are relatively better implemented in some districts than in others.

**Results:**

The findings indicate that in Lindi district the pro-poor exemption mechanism was ineffective in implementation. There were no clear ways of identifying and protecting the poor household members. In contrast, in Iramba district the policy was relatively better implemented. The poor were identified at the village, ward, health facility and district levels. In some villages, the poor were grouped in 10s to form one household. Then, using the village funds, the Community Health Fund cards were purchased for them. Personal initiatives of the key district leaders, commitment of the district health management team and local government officials, regular supervisory visits, as well as incentives to the health facility committees and boards were the main factors that facilitated the implementation of the pro-poor exemption policy.

**Conclusions:**

It is concluded from this study that management and leadership practices including personal initiatives of the key district leaders, effective supervision mechanisms, commitment of the district health management team and local government officials, as well as incentives for the health facility committees and board members are pivotal for the implementation of the pro-poor exemption policies.

## Introduction

Equitable access to primary health care is vital to the overall health and development of a country. Tanzania, like many other African countries, has been implementing user fee policy in its health sector since the early 1990s. User fees were seen as an effective solution for funding improved health care. However, studies have shown that user fees contributes to catastrophic expenses [1] and reduce people’s access to health services especially the poor and the most vulnerable groups of the society [2-4]. Accompanying user fee, mechanisms were designed to protect and ensure equitable access to health care to poorer segments of the population. In Tanzania, the Ministry of Health introduced waivers and exemptions in 1994, to ensure access of health services by the poor and vulnerable members of the society. Exemptions are statutory entitlements that are automatically granted for all maternity services, children under five years and particular diseases such as TB/Leprosy, HIV/AIDS and some chronic diseases that would drain substantial income from the patients if such patients were asked to pay [5,6]. Waivers on the other hand target the poor and vulnerable groups of the society on grounds of ability to pay [5].

The exemption and waiver system was expanded with the introduction of the Community Health Fund (CHF)^a^ scheme which aimed at improving access to health care and to protecting people against financial cost of illness in an environment with shrinking budgets for the health sector. In the framework of the Community Health Fund, districts are supposed to pay the premiums of the poorest households. According to the Community Health Fund Act the power to issue an exemption from the Community Health Fund payment is vested within the Ward Health Committee upon receiving recommendations from the Village Council. The Village Council will then issue a Community Health Fund membership card to the identified households. The Act further requires the exempting authority (local governments) to seek alternative means of compensating the Fund [7].

In Tanzania, a body of evidence indicates that exemptions policies, while potentially effective in principle, are ineffective in implementation [8-14]. Munyetti and colleagues [9] reported that only 16% of patients who were unable to pay for health services were exempted from paying user fees. The remaining 84% either borrowed money to pay for health care, self treated or sought traditional health care. Another study found out that while exemptions were given to a relatively large number of people (children under five years of age, pregnant women, the chronically ill and those considered being too poor), the most needy did not benefit such exemption [10]. In addition, communities and health workers alike reported lack of information and understanding of the exemption scheme; thus the policy was interpreted differently by different facilities, and record-keeping and transparency were poor [10]. Similarly, other studies have reported that most district managers do not provide exemptions, for fear of jeopardising the Community Health Fund’s financial viability [11-14].

In contrast, there is evidence that some districts, despite the challenges, perform better than others in terms of identifying the poor and allocating funds for the poor and vulnerable groups [8,15-18]. For example, Mwanga district was reported to have made efforts to identify the poor and maintain lists of the poor in all health facilities so that the application of waivers was straight forward [8]. In Muheza, a number of criteria defining the poor to be waived from Community Health Fund membership contributions had been defined, including elders and widows who had no means of support, physically and mentally handicapped persons, orphans less than 18years and those with poor housing and had no access to safe drinking water [15]. Qualifying individuals had to apply for pro-poor funding access to the village council, and had to be seconded by hamlet members [15].

However, there are as yet very few studies on the implementation of exemption policies for the poor and vulnerable members of the society. More importantly, there is scant knowledge on why exemption policies are implemented better in some districts than in others. Against this backdrop, this study aimed to explore why exemption policies in Tanzania are relatively better implemented in some districts than in others.

### The structure of the district health system in Tanzania

In the 1990s, the Tanzanian government introduced a series of health reforms including decentralisation. The decentralisation policy in Tanzania has led to a major transformation from a centralised administrative system to a decentralised structure where local governments assume a greater role in the provision of health services. In the district, the full council is the highest political body and has overall authority for all district health services. Under the full council is the Council Health Services Board (CHSB) which is responsible for overseeing all health services delivery in the district. The CHSB consists of four members from the community. Below the CHSB is the Council Health Management Team (CHMT) which is responsible for planning and budgeting for activities needed to manage, control, coordinate and support all health services in the district on a year-to year basis. Other responsibilities of the CHMT include: ensuring implementation of health activities by hospitals, health centres, dispensaries, and communities; and to monitor and evaluate implementation of health activities in the district. Below the CHMT are the health facility committees which are tasked with strengthening community-level decision making about facility matters, including fund-related decisions. The health facility committees are also a mechanism for the community members to get involved in advocating enhanced service delivery [19].

## Methodology

### The study design and settings

An exploratory case study design focusing on two districts - one relatively high performing (Iramba) and the other fairly low performing (Lindi) - was adopted [20]. The ‘high’ and ‘low’ performing categorisation was based on the enrolment of the Community Health Fund^b^. However, the districts were not selected based on Community Health Fund performance only; other factors such as geographical accessibility were also taken into account. Table 1 summarises the key characteristics of the study settings.

**Table 1:** Key characteristics of the study settings

	**Iramba district**	**Lindi district**
Population	236,282 people	194,143 people
Community Health Fund enrolment rate^1^	28.1%	0.4%
Hospitals	2	1
Health Centres	6	6
Dispensaries	60	30
Divisions	7	9
Wards	31	30
Villages	143	134
Health workers available	43%	50%
Shortage of health workers	57%	50%

^1^The national Community Health Fund coverage rate is about 7.9% (National Health Insurance Fund data of 2011).

In each district, one hospital, two health centres and four dispensaries were purposively selected in consultation with the district medical office (See Table 2). The health centres and dispensaries were selected based on a number of criteria including geographical accessibility and localisation, and community health fund enrolment.

**Table 2:** The sampled study sites

	**Name of the facility**	**Type of the facility**	**District**
1	Kiomboi	Hospital	Iramba
2	Ndago	Health Centre	
3	Kinyangiri	Health Centre	
4	Bomani	Dispensary	
5	Mampanta	Dispensary	
6	Misigiri	Dispensary	
7	Ulemo	Dispensary	
8	Kitomanga	Health Centre	Lindi
9	Rutamba	Health Centre	
10	Mchinga	Dispensary	
11	Kilangala	Dispensary	
12	Mahumbika	Dispensary	
13	Mnolela	Dispensary	

### Data collection techniques

The study was primarily based on the individual interviews with the key stakeholders. Documents and health facility visits were used to support, verify and highlight the key issues that emerged. In Iramba district, individual interviews were carried out in October 2012 while in Lindi the study was conducted in January 2013. Each interview lasted approximately 45 minutes and was carried out at the respondent’s workplace and/or home. In order to cover a wide range of views of different actors, a purposive sampling technique was used. At the district level, members of the Council Health Management Team and the Council Health Service Board were interviewed. At the health facility level, committee members at the district hospital and health centres were interviewed. In total, 83 interviews were carried out (51interviews in Iramba and 32 interviews in Lindi) (see Table 3). Furthermore, considerable documentary information was obtained and the analysis was validated with observations at the health facilities.

**Table 3:** Categories of key informants

**Category of respondents**	**Number interviewed**	
	**Iramba**	**Lindi**
Council Health Service Board	4	3
Council Health Management Team	3	3
Hospital Governing Committee	3	-
Health Centre and Dispensary Committees	17	15
Councillors	2	-
Village Executive Officers & Village leaders	8	4
Heads of health facility & health workers	12	7
Social Welfare Officers	2	-
**Total**	**51**	**32**

### Data analysis

Quantitative data that were recorded in numerical form in the documents reviewed were entered into an Excel worksheet for secondary analysis of totals, percentages and other such simple statistics as necessary. Thematic approach was adopted to analyse qualitative data [21]. First, all the interviews were transcribed into Kiswahili verbatim. Second, the transcriptions were translated into English. Third, interview transcripts were entered into Nvivo 10 software for storage, coding, text search, and retrieval. A list of themes was drawn up, based on the objectives of the study and observations from the field. Using Nvivo 10 software, data were coded to initial themes. Thereafter, data were sorted and grouped together under patterns that were more precise, complete, and generalisable. As patterns of meaning emerged, the researcher searched for similarities and differences. Finally, data were summarised and synthesised, retaining as much as possible key terms, phrases and expressions of the respondents. After this analysis, data were triangulated to allow comparison across different categories of respondents for final interpretation and presentation of results.

### Ethical issues

The research was approved by the University of Dar es Salaam which has been given mandate to issue research clearance to its staff and students on behalf of the Government of Tanzania and Tanzania Commission for Science and Technology. The clearance was presented to the regional and district authorities who approved the study in their administrative areas. Verbal consent was sought from prospective informants after explaining the objectives of the study. Informants were informed of the right to withdraw from the study any time they wished. They were also assured of confidentiality of any information deemed necessary to be treated so. All interviews were tape recorded with the consent of participants and the resulting recordings and transcripts were stored in a manner that protected confidentiality.

## Results

The following section presents the main findings of the study organised in three major themes: the process of exempting the poor; innovations to improve the performance of the pro-poor exemption policy; and challenges constraining the implementation of the pro-poor exemption policy.

### How were the eligible poor identified?

In Lindi district, the data revealed contradictory views among the district health officials and members of the board. The vast majority of the district health officials reported that exemptions were granted to elders at 60 years of age and above. They reported that the village executive officers wrote a letter to introduce the eligible poor to the health service providers. Similarly, the majority of in-charges of the health facilities and health workers confirmed that elders who were unable to pay came with an introduction letters from the village executive officers. Further, a few members of the health facility committees and health workers reported that sometimes elders were identified by the health providers at the point of service delivery.

In contrast, a few district officials were unaware of the existence of the exemption policy. When asked what criteria were used to identify the poor, one respondent remarked this way:

“*There is no exemption policy*. *However*, *we have heard in the political platforms that elders at 60 years of age and above are supposed to get free health services*. *Therefore*, *the CHMT suggested that all elders who need exemption should be identified in their respective villages and should be given an official letter to enable them get free health services*” (IDI with a CHMT member).

Similar views were reported by a few members of the health facility committees. When asked if there were individuals who had been granted exemption, one respondent illustrated this way:

*I have not heard it officially*. *But I just heard that the district has a plan of indentifying the poor and issue CHF membership card*. *At this facility*, *I have not seen anyone who has been granted exemption*” (IDI with a committee member).

Further, in all health facilities in Lindi district which the researchers visited, there was no any record of the poor who had been granted exemption. The researcher, however, managed to see records of individuals who were entitled to statutory exemptions such as pregnant women and children under five years of age. Interviews with the district health officials in Lindi district revealed that the district plans to start identifying the poor household members and issuing Community Health Fund membership card from the 2013/2014 financial year. However, the process had not started and it was not clear how it would be implemented at the village and ward levels.

On the other hand, in Iramba district, there was unanimous agreement among district level respondents that two different approaches were used to identify the poor. First, the poor who needed exemption presented themselves to the village executive officers. The village leaders assessed the individuals and issued a letter for the poor to access health services at the dispensary and health centres. In case the poor needed referral to the district hospital, the village leaders wrote a letter to introduce the poor to the district social welfare officer. The district social welfare officer assessed the candidate and wrote a letter which was then presented to the district hospital. At the district hospital, there was a hospital social welfare officer whose role was to assess and keep records of all individuals who have been granted exemptions.

Similar to the above, most interviewees from the facility and village levels in Iramba district confirmed that the poor were identified by the village and ward leaders and the list was submitted to the health facility committee. Some respondents remarked as follows:

“*Village leaders are informed to identify the eligible applicants in their respective areas*. *Names are identified at the street* (*Mtaa*) *level and are forwarded to the Ward Development Committee for approval*. *Then having been approved at the ward level*, *the names are submitted to the health facility committee*” (IDI with a committee member).

Another respondent added:

“*The village councils identify names of the qualified members*. *The names are brought to the Ward Development Committee* (*WDC*). *At the WDC*, *we normally scrutinise the names*. *We want to ensure that only individuals who deserve are granted exemption*. *Of recent*, *there has been an influx of elders who need exemption*. *Therefore*, *we normally grant exemption to only those who are unable to pay*” (IDI with a Councillor).

Respondents, in Iramba district, reported a number of criteria that were used to identify the poor including those who could not sustain themselves with enough food and had no relatives, persons over 60 years who could not pay for their health care costs, and those who could not produce due to disability. However, there were indications from the data that these criteria were not applied consistently.

In Iramba district, the researchers managed to interview social welfare officers, one based at the district hospital and another at the District Executive Officer’s office. Both interviewees confirmed that exemptions were provided to the poor, vulnerable groups and elders at 60 years of age and above who could not pay for themselves.

Furthermore, in Iramba district, the researchers managed to see a sample of letters which had been written by the village and ward leaders to introduce the eligible poor members of the community to the district social welfare officers. Table 4 shows the number of exemptions for elders of 60 years of age and above reported to the social welfare officer at the district hospital in Iramba district from 2009 to 2012.

**Table 4:** Exemptions for elders 60 years of age and above from 2009 to 2012

**Year**	**Number of exemptions**		
	**Male**	**Female**	**Total**
2009	39	64	103
2010	48	75	123
2011	44	64	108
2012	74	110	184
**Total**	**205**	**313**	**518**

In Iramba district in all health facilities which the researchers visited had a list of the exempted people on the notice boards inside the office of the person in-charge for the health facility. In addition, it was evident in almost all facilities that exemption was one of the permanent agenda in health facility committee meetings. The health facility committees in Iramba district held meetings monthly to discuss various issues pertaining to the performance of the health facilities. Records of the meetings were available in all health facilities which the researchers visited. Two factors made it possible for the committees to hold their meetings as required. First, the Iramba district council had managed to ensure that the health facility committee members were paid incentive to compensate their time. The chairperson and the secretary of the committee were paid TShs 3,000 (equivalent to 2 USD) while other members of the committee were paid TShs 2,000 (equivalent to 1.5 USD) after attending every meeting. Second, the CHMT had established regular supervision and monitoring system which required all health facilities in the district to submit minutes of the meetings along with other reports to the CHMT on a monthly basis, from 1st to 6th date of every month. The District Medical Officer’s office provided travelling allowance for health workers who submitted reports. Almost all in-charges of the health facilities viewed this as an incentive that motivated them to submit the reports and the meeting minutes on time.

By contrast, in Lindi District, the committees held their meetings quarterly. The chairpersons and secretaries of the health facility committees were paid TShs 15,000 each (equivalent to 10 USD) while other members were paid TShs 10,000 (equivalent to 7 USD) per meeting. However, analysis of the interviews across all respondents revealed that sitting allowances were hardly paid or were paid very late, sometimes even after three or four meetings (three to four quarters). There were indications from the data that in Lindi district some members of the committees were not attending the meetings as regularly as required.

### What challenges constrained the implementation of the pro-poor exemption policy?

Data from both districts revealed a number of challenges that constrained the effective implementation of the exemption policy. Overall, there appeared to be confusion about the eligibility criteria for granting exemption to elderly group. The confusion created variations in the implementation, potentially hampering the effectiveness of the pro-poor exemption policy. Some respondents remarked this way:

“*The policy is blind and the government has been silent on this despite its importance*. *The government has just said elders above 60 years should be granted exemption*” (IDI with CHMT member in Lindi district).

Another responded added:

“*There is confusion*. *The policy says exemption should be granted to elders who are 60 years and above and are unable to pay*. *But the policy is interpreted differently*. *People think that all elders*, *regardless of their economic status*, *deserve free services*. *So*, *there are two things*: *policy and politics*. *To a large extent*, *politics seem to be stronger than the policy*” (IDI with CHSB member in Iramba district).

Similar views were reported at the facility level. As a result, everybody was implementing the policy in his own way. One respondent commented this way:

“*The main challenge is that people have different interpretations of the policy*. *When district health officials come for supervision they tell us different issues*. *For example*, *one official may tell you that elders should be given free services*. *Another official may insist that they are supposed to be given a CHF card paid by the village government*. *So*, *we are confused and we do not know which one is correct*…” (IDI with in-charge of health facility in Iramba district).

Another common challenge which was reported by almost all respondents was increased financial burden to the health facilities. In Iramba district, respondents reported increased number of elders who demanded exemption. This was attributed to the Prime Ministers’ speech on marking the world elders’ day on 1st October 2010. The Prime Minister ordered all districts in Tanzania to ensure that all elders 60 years of age and above are provided free health services regardless of their ability to pay. Consequently, health facilities were unable to cope with the increased demand for free health services.

“*At this hospital*, *we have done an assessment and discovered that over 50 percent of patients come from the exempted groups*. *This is a big challenge*, *not only at this hospital*, *but also in other facilities in the district*. *Although people do not want to be told the truth*, *our health facilities cannot afford to bear this burden*” (IDI with CHMT member in Iramba).

Closely linked to this, in both districts of the study, the majority of respondents did not see the rationale of providing free health care to all elders 60 years of age and above. They pointed out that some elders were better-off and could afford to pay the Community Health Fund contributions. One respondent put it this way:

“*Life expectancy has increased*, *people turn 60 and they still look young*. *For example*, *in the next seven years I will turn 60*. *So*, *do you want to say I should also get exemption*? *I will not be fair because the facility will be losing and I will be jeopardizing chances of individuals who are really unable to pay*” (IDI with a CHMT member in Iramba).

Furthermore, in both districts of the study almost all respondents reported that frequent drug stock-out at the facilities constrained the implementation of the pro-poor exemption policy. There was unanimous agreement across all categories of respondents that sometimes individuals who had been granted exemption were required to purchase drugs in the private pharmacies. Drug stock-out was for the most part attributed to the inadequate and late supply from the Medical Stores Department (MSD). The majority of respondents had the opinion that the medical stores department lacked the capacity of supplying drugs to all facilities. They suggested the possibility of diversifying the drug procurement system in the country by opening opportunities for other drug suppliers. However, there were indications from the data that drug stock-out problem was more severe in Lindi than in Iramba district. According to the data, Iramba district often procured supplementary drugs using Community Health Funds. In addition, Iramba district had established an effective supervision and management of drugs within the facility. The CHMT conducted frequent drug audit in all facilities in order to control irrational use of the drugs by the health providers. This eventually reduced the rates of drug stock-out in the facilities.

### Innovations to improve the performance of the pro-poor exemption policy

In Iramba, all district health officials and members of the board reported that the CHMT had discovered that approximately 50 percent of the patients at the district hospital were from the groups which had been exempted from paying user fees. The CHMT, through the District Medical Officer, advised the District Executive Director on the need to control exemptions. The CHMT members proposed the idea of grouping the poor elders in the groups of ten people to form one household. Then, using the village funds, the CHF cards were purchased for them. In order to implement this decision, the District Executive Director directed all villages to identify eligible elders and purchase the CHF membership cards. In this new approach, the village councils were responsible for identification and purchasing of the CHF membership cards. The elders were given freedom to decide among themselves who should keep the CHF membership card. The district council, therefore, benefited from the matching grant provided by the central government to all CHF beneficiaries.

In some villages which the researchers visited in Iramba district, village leaders confirmed that they had been paying CHF premiums for the elders who were unable to pay. Funds came from the village own generated sources. However, the data revealed that not all villages managed to effectively implement this new approach of protecting the poor from the medical impoverishment. A few respondents, particularly at the village and ward levels, raised concerns regarding the sustainability of this pro-poor funding mechanism. Nevertheless, the majority of district level respondents generally felt that this was a viable approach of implementing the pro-poor exemption policy given resource constraints in the districts.

## Discussion

This paper has described the experiences of the districts and local level actors in implementing pro-poor exemption policy in Tanzania and illustrates a number of factors for the diverse implementation of the policy in the two study-districts. The approach adopted is broadly descriptive, seeking to provide in-depth analysis of the factors that influence the implementation of the pro-poor exemption policy in Tanzania. In this section, the most important issues raised by the respondents are further discussed with reference to wider literature on the implementation of health policies in developing countries.

The findings indicated that in Lindi district the pro-poor exemption mechanism was ineffective in implementation. There were no clear ways of identifying and protecting the eligible poor household members. In contrast, in Iramba district the policy was relatively better implemented. The poor were identified at the village, ward level, health facility and district level. The list of the poor was available in almost all health facilities. In some villages, the poor were grouped in the groups of 10 to form one household and were issued a Community Health Fund membership card purchased by the village governments.

Although, the study did not interview beneficiaries who have been granted exemption in order to assess their experiences and perceptions on the health services provided in line with exemption policy, it could reasonably be assumed that exemptions provided opportunities for the poor and vulnerable groups access to services which they would otherwise not have access if such patients were asked to pay. Evidence indicates that user fees is the major barrier of access to health services for the poor and most vulnerable groups of the society [1-4]. However, equitable access to health services may be influenced by other factors such as distance, availability of the services, socio-cultural and health system factors. More scientific efforts are needed to explore the extent to which exemptions and waivers provide more equitable access to health services for intended service users.

The findings suggest that effective supervision and commitment of the district health management teams were the important factors for the better implementation of the exemption policy in Iramba district. The large variation in the policy implementation suggests that in Iramba, district health managers, particularly district medical officer and district executive director, were able to initiate several innovations for the implementation of the pro-poor exemption policy and it is largely their personal initiatives that have determined how the policy took shape on the ground. This is in line with the findings of previous studies showing that the district health managers were the main factor for the poor performance of the exemption policies [12,13]. Studies in other contexts have shown that the district health managers and local level officials who are allowed wider discretion may choose not to take advantage of the new powers and simply continue to pursue activities as they had before [22,23]. Alternatively, they may choose to innovate by making new choices they had not made before thereby improving the performance of the policies [22-24].

It was evident from the findings that there were some confusion about the eligibility criteria for granting exemption to elderly group. The failure of the central government to define eligibility criteria for waivers, compounded with limited technical support, may have exacerbated problems and contributed to the variation in the implementation of the pro-poor exemption policy between districts. Previous studies have shown that lack of knowledge regarding a reform, and its intended beneficiaries can result in low motivation and commitment of health system actors and can impede implementation [12,25]. Likewise, poor dissemination of policies can lead to confusion and variations in what is implemented [12,26-28]. Improving communication strategies to inform the policy implementers and the general population has been identified as one of the key points in the implementation literature [29,30]. In this regard, the Ministry of Health and Social Welfare should devote more time to explain and communicate the eligibility criteria for the pro-poor exemption, particularly for elderly group of the society. This would improve awareness and understanding of the policy on the part of the policy implementers and the wider public. Policies need to be socially acceptable to the groups affected and ideally policy implementers, including the public, should be involved in the formulation process [31].

The findings, further, suggest that financial incentive to the health facility committees and board members was the main factor that facilitated the good performance of the pro-poor exemption policy. In Iramba district, the district health managers had in essence managed to ensure that the health facility committee members were paid incentives on time. This motivated members of the health facility committees to hold their monthly meetings as required. The fact that the health facility committees held their meeting monthly made it possible to frequently discuss the implementation of the exemption policy in their respective health facilities. In contrast, in Lindi district, while the health facility committees were in place, meetings were held quarterly and the majority of the members were demoralised due to the late and or non-payment of the sitting allowances.

While in Iramba district exemption policy was relatively better implemented than in Lindi district, it is evident that in both districts, there were no routine processes of identifying the poor before they fall sick. Although in Iramba district a few elders were identified and issued with CHF membership cards, for the most eligible poor, the identification was largely being done when the person was sick. These findings suggest that the effectiveness of this process mainly depends on the seriousness of illness. If the person is seriously ill it is possible that this person will directly go to the facilities and pay to get treated instead of going the long route of presenting themselves to the village executive. Further, the fact that the poor had to seek exemption from the village leaders means that those who were not aware of the exemption policy were unlikely to benefit from the policy. The districts need to institutionalise the process of identifying the eligible poor prior to illness. Exemptions and waivers should follow the poor and not the vice versa. The village and ward leaders should be empowered to identify the poor based on clearly defined eligibility criteria. It is evident that communities know each other better and this might make the identification process easy. The list of the poor could be updated annually or as need arise.

Closely linked to this, while it was evident that in Iramba district the poor, mostly elderly people, were being identified, a vast majority were not being issued a CHF membership card, but instead an exemption letter which granted them free care at the health facilities. While this practice addresses the issue of supporting those who are unable to pay, it may also stigmatise the household by labelling them as poor instead of allowing them to blend in with all of the other card holders.

Furthermore, it is evident from the analysis that the health facilities in Tanzania are unable to cope with the increasing demand for exemptions. While there were no systematic records of how many exemptions and waivers were granted in which categories, in Iramba district the CHMT estimated that around 50 percent of health services provided were exemptions or were provided to people who have a waiver. Collection of user fees in the government health facilities was therefore minimal. While it is beyond the scope of this study, the increased demand for exemption may have serious impact on the quality of services and health systems. A recent review indicated that while exemptions increased utilisation of health services, it had destructive effects on health systems [32]. Given the fact that the local government authorities in Tanzania are financially over dependent on the central government, the implementation of the pro-poor exemption will likely continue to face challenges. In this regard, the poor people will need to be subsidised from pooled funds, generally government revenues. Such assistance could take the form of direct access to government-financed services or through subsidies on their insurance premiums.

### Limitations of the study

This study relied primarily on the review of minutes, health facility visits, and key informant interviews with the community representatives and the district health managers. First, the study did not interview beneficiaries who have been granted exemption as well as community members in order to assess their experiences and perceptions on the health services provided in line with exemption policy. Secondly, the study was limited to two districts due to budget and time constraints. While efforts were made to sample respondents from different levels of decision-making in the district, the sampling strategy does not allow for generalisation of the results. However, the objective of this study was not to make statistical inferences but to understand in depth the variation in the implementation of the pro-poor exemption policy between districts. Notwithstanding these limitations, the study provides good insights into factors for the diverse implementation of the pro-poor exemption policies in Tanzania.

## Conclusions

It is concluded that Iramba district has fundamentally managed to design a number of innovations that facilitated better implementation of the pro-poor exemption policy. Personal initiatives of the key district leaders, commitment of the district health management team and local government officials, effective supervision mechanisms, as well as incentives for the health facility committees and board members were the main factors that facilitated implementation of the pro-poor exemption policy in Iramba district. These findings reaffirm the need to manage and coordinate numerous actors involved in the implementation of the policies. Furthermore, given the fact that the local government authorities in Tanzania are financially over-dependent on the central government, the implementation of the pro-poor exemption will continue to face challenges. The poor people will need to be subsidised from pooled funds, generally government revenues. Such assistance can take the form of direct access to government-financed services or through subsidies on their insurance premiums.

## Endnotes

^a^The CHF is a district-level voluntary prepayment scheme, introduced in parallel with user fees at public health facilities, that targets the population living in rural areas and/or employed in the informal sector.

^b^Community Health Fund performance data were obtained from the Budget speech of the Ministry of Health and Social Welfare 2011/2012.

## Competing interests

The authors declare that he has no competing interests.
